# THE ROLE OF WORKPLACE POLICY ON EMPLOYED FEMALE CAREGIVERS DURING THE CORONAVIRUS PANDEMIC

**DOI:** 10.1093/geroni/igac059.632

**Published:** 2022-12-20

**Authors:** Jessica McLaughlin

**Affiliations:** Knoebel Institute for Healthy Aging , Denver, Colorado, United States

## Abstract

The coronavirus pandemic has indelibly impacted society over the past two years. This qualitative study explores how working female caregivers, in particular, experienced the pandemic and how the workplace supported them during this time. Findings from interviews with 29 working female caregivers revealed that many caregivers were unable to set boundaries around caregiving during the pandemic. Caregivers frequently found themselves on their own in providing care. This meant that caregivers had little time to themselves to rest and recharge. Whereas prior to the pandemic, caregivers may have had help with caregiving through services like respite and adult daycare, these services were no longer options. This reduced level of external support and care influenced caregivers’ socioemotional wellbeing. Workplace policies, such as the ability to work remotely and working flexible hours, helped to ease caregiver burden. These findings have implications for both caregivers and workplaces during future crises and disasters.

